# Treatment of Femoral Neck Fractures: Unipolar Versus Bipolar Hemiarthroplasty

**DOI:** 10.5704/MOJ.1307.007

**Published:** 2013-07

**Authors:** Somashekar, Sathya Vamsi Krishna, JN Sridhara Murthy

**Affiliations:** Department of Orthopaedics, Kempegowda Institute of Medical Sciences, Bangalore, India; Department of Orthopaedics, Kempegowda Institute of Medical Sciences, Bangalore, India; Department of Orthopaedics, Kempegowda Institute of Medical Sciences, Bangalore, India

## Abstract

Background and objective:Intracapsular femoral neck fractures are common in the elderly population. To avoid the poor outcome of internal fixation and for early mobilization, hemiarthroplasty is performed. However, there is inadequate evidence to support the choice between unipolar or bipolar hemiarthroplasty. The aim of this study was to compare the outcome of unipolar with the bipolar prosthesis in geriatric patients. Methods: Forty-one patients above 60 years of age and an acute displaced fracture of the femoral neck were randomly allocated to treatment by either unipolar or bipolar hemiarthroplasty, in the Department of Orthopaedics, between September 2009 and October 2012. Functional outcome was assessed and compared using Harris hip score and radiological parameters with a follow-up of one year. Results: The two groups of patients with mean age of 67.3 in bipolar group and 75.6 in unipolar group did not differ in their pre-injury characteristics and perioperative parameters. The mean Harris hip score in bipolar and unipolar groups was 86.18±12.18 and 79.79±15.55, respectively (p=0.183); range of motion was 210.63±28.39 and 181.58±37(p=0.015) with bipolar and unipolar groups, respectively. Functional activities were better in the bipolar group. Complications like painful hip, posterior dislocation, periprosthetic fracture and acetabular erosion were encountered in unipolar prostheses. Conclusion: The use of bipolar endoprosthesis in the management of displaced femoral neck fractures in the elderly was associated with better mean Harris hip score and incidence of complications was limited. Hence, bipolar would be a better option in elderly patients with fracture neck of femur.

**Key Words:**

Unipolar; Bipolar; Hemiarthroplasty

## Introduction

Fracture of the hip is a common injury. With increasing life expectancy worldwide, the number of elderly individuals is increasing, and it is estimated that the incidence of hip fracture will rise from 1.66 million in 1990 to 6.26 million by 2050. According to the Swedish National Hip Fracture Register, intracapsular fractures of the femoral neck constitute 53% of all hip fractures with 33% undisplaced and 67% displaced^1^.

The rationale for operative treatment by means of internal fixation is to reduce the risk of secondary displacement from undisplaced and displaced fractures, and to maintain fracture reduction for displaced fractures. The main reasons for the failing of internal fixation are avascular necrosis and nonunion. Failure of internal fixation leads to a re-intervention rate of 35% with decreased function and increased morbidity as demonstrated by a meta-analysis by Lu Yao 2.

Replacement of the femoral head and neck with a prosthesis offers a way to prevent complications of internal fixation and is therefore an attractive alternative in the elderly patient^3^. There is however no consensus on how to treat patients with a displaced intracapsular fracture between sixty and eighty years of age. It is because of the poor clinical results that the displaced intracapsular fracture is referred to as "the unsolved fracture"^4^.

Moore and Bohlman^5^,^6^ after removal of a giant cell tumor of the femoral head, introduced hemiarthroplasty in 1940. Since then it has also been used for the treatment of displaced femoral neck fractures. It had the following features: solid polished unipolar head with a collared, straight, fenestrated stem designed for non-cemented use.

The development of bipolar hemiarthroplasty was based on the clinical experience with limited success of unipolar prosthesis due to progressive acetabular erosion and protrusion.

Based on Charnley’s pioneering arthroplasty principles, two bipolar designs emerged in the early 1970’s: the Bateman and the Gilberty prostheses.

This is a prospective randomized study of the short-term results of hemiarthoplasty using Austin Moore unipolar prosthesis and bipolar prosthesis.

Outcomes at six weeks, three months, six months and 12 months were analyzed and compared using Modified Harris hip score and radiographs.

## Subjects and Methods

**Fig. 1:**
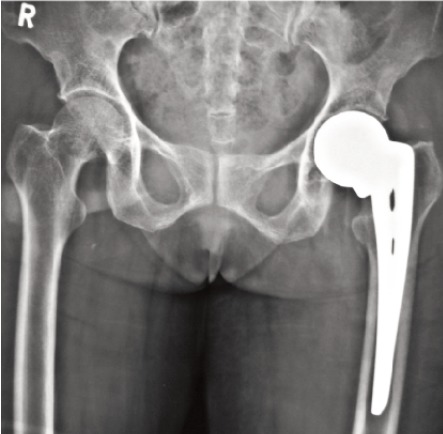
: Post operative radiograph of unipolar prosthesis

**Fig. 2:**
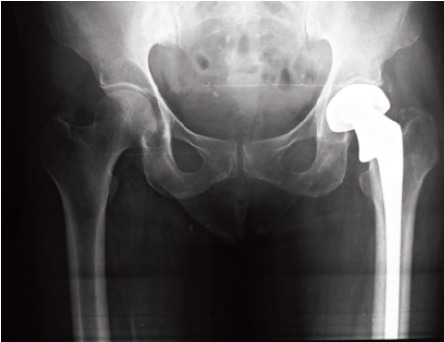
: Post operative radiograph of bipolar prosthesis.

The present study is of intracapsular fracture neck of femur in elderly patients above the age of 60 years, irrespective of gender, treated with hemiarthroplasty using uncemented unipolar Austin Moore’s prosthesis (AMP) in 20 patients and bipolar endoprosthesis in 21 patients, in the Department of Orthopaedics at Kempegowda Institute of Medical Sciences (KIMS), Bangalore, selected on the basis of purposive sampling (judgment sampling) method (Figure 1 and 2). All implants used were manufactured by Inor Medical Products, Mumbai, India. All the patients were walking normally before injury.

All patients were operated through a southern approach, and received antibiotics and venous thromboembolism prophylaxis. Postoperatively, full weight bearing was allowed with the help of physiotherapists as per their compliance. The patients were assessed preoperatively and post operatively based on Harris hip score at intervals of six weeks, three months, six months and one year. Sequential radiographs were compared to assess diminishing joint space, acetabular erosion, proximal migration and protrusion of the acetabulum. Loosening, subsidence and angular shift of the femoral stem were also assessed on these radiographs Descriptive and inferential statistical analyses were carried out in the present study with Student t test (two tailed, independent), inter group analysis on metric parameters. Chi-square/ Fisher Exact test were used to find the significance of study parameters on categorical scale between two or more groups. Ethical clearance was obtained from our institutional ethics committee.

## Results

Patients who had unipolar prostheses were comparatively older to those with bipolar prostheses (75.5 vs. 67.3, P<0.01). Females constituted 65.8%. Mortality rate was statistically similar in both groups, due to age related factors (p=0.663). Mean length hospital stay was similar in both groups (p=0.894).

All cases were analyzed based on the Harris hip score (Table I). The total score was tabulated and graded as excellent, good, fair, poor and failure (Table II).

**Fig. 3:**
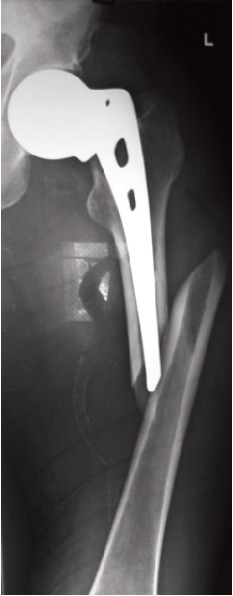
: Periprosthetic fracture of unipolar prosthesis.

**Fig. 4:**
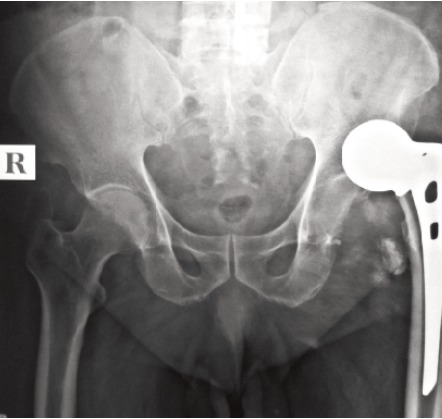
: Posterior dislocation of unipolar prosthesis.

**Fig. 5:**
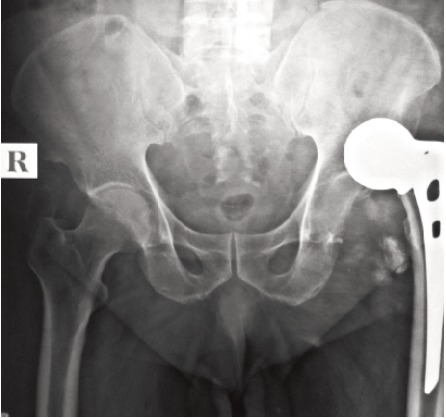
: Acetabular erosion in unipolar prosthesis.

Most of the complications were recorded with the unipolar group (Table III). All cases, one (4.7%) in the unipolar group presented with posterior dislocation (Figure 4) on the 8th post operative day, for which closed reduction was done under GA and immobilized for one and half months and there after mobilized successfully. Another case (4.7%) of unipolar group presented with periprosthetic fracture (Figure 3) after three months following trauma, which was managed with open reduction and internal fixation with plate and screws retaining the same prosthesis. The patient was mobilized after two months and he continued to have thigh pain. A case of acetabular erosion (Figure 5) was noted in the unipolar group. A single case of superficial infection was recorded in each group, which responded to antibiotics.

## Discussion

Comparison between 21 cases of bipolar hemiarthroplasty and 20 cases of Austin-Moore prosthetic replacement for femoral neck fractures in elderly patients over a one year period has shown that patients with bipolar prostheses had better functional outcomes in terms of range of motion, ability to use public transport and ability to cut toe nails. Mean Harris hip score was better with the bipolar group.

Lunceford Jr7 felt that the pain following hemiarthroplasty should not be the reason for condemning the procedure. He listed the following causes for pain: infection, improper prosthetic seating, metallic corrosion and tissue reaction, improper sized femoral head, contractures, periarticular ossification, toggle or acetabular wandering and redundant ligamentum teres.

Limping is a common consequence of hemiarthroplasty in adults. Alteration in the abductor mechanism due to a marginally greater excision of neck is the most probable cause^8^.

Cornell et al^9^ reported that patients with bipolar prosthesis did better on walk tests and had better range of motion at six months. Sabnis and Brenkel10 reported 14 % unipolar patients walking unaided compared to 54% of bipolar patients walking unaided.

Yamagata et al^11^, in their classical study, reviewed 1001 cases of hip hemiarthroplasty. There were 682 unipolar and 319 bipolar cases. Patients undergoing bipolar arthroplasty exhibited higher hip scores and lower acetabular erosion rates compared to the unipolar replacement.

Lestrange^12^ reviewed 496 patients with bipolar replacement for displaced femoral neck fractures and compared them with patients having fixed-head prosthesis. He found that the bipolar prosthesis offered advantages over one-piece designs in terms of stability, decreased acetabular erosion and improved function.

D’Arcy and Devas^13^ reported incidence of dislocation following prosthetic replacement ranging from 0.3% and 10%.

Dislocation following hemiarthroplasty was due to the disruption of the posterior stabilizers while performing the posterior approach, ultimately leading to failure and dislocation^14^. The dislocated hemiarthroplasties have a lower center-edge angle of Wiberg and the patients with low offset hips were more inherently unstable and hence prone to dislocation. The posterior approach is associated with higher dislocation rate^15^. Sikorski and Barrington^16^ reported dislocation rates of 10% in the unipolar prosthesis. Blewitt and Mortimore^17^,^20^ reviewed cases of dislocation in a series of 1000 consecutive hemiarthroplasties. Recurrent dislocation can be related to component malalignment or improper soft tissue tensioning.

Bochner et al^18^ observed that dislocation occurs less frequently with bipolar prostheses. The theoretical advantage of the bipolar prosthesis is that the combined arc of motion of the dual joint should reduce the incidence of dislocation, because most of the motion during activities of daily living should take place at the inner articulation. Attarian^19^ reported that bipolar prosthesis has a self-aligning acetabular component, which finds a correct orientation on its own (self-centering mechanism), and the incidence of subluxation and dislocation is low.

Whittaker et al^20^ reporting in a series of 160 hemiarthroplasty cases noted the rate of joint spacing in a 5-year study was 64% with the unipolar prosthesis. Acetabular erosion occurs as a result of impact causing injury to the acetabular cartilage at the time of the trauma, especially as the elderly often sustain injury by a fall directly on the hip.

Excessive pressure on the acetabular cartilage after arthroplasty also produces erosion when insufficient femoral neck is resected. The exact matching of the size of the prosthetic head is particularly important as too large a head produces ring wear of the acetabulum and too small a head increases point bearing with subsequent wear. Finally, the cemented metal implant within the upper part of the femoral shaft will be more likely to transmit the impact of each step with greater stress across the prosthesis to bone interface than would normal bone in which there is considerable resilience^21^.

Skala-Rosenbaum et al^22^ observed that prosthesis migration depended on the position of the head, CE angle and position of the prosthetic stem in the medullary canal. The resection level of the femoral neck and the subsequent position of the prosthetic head is a significant factor influencing the progress of acetabular erosion.

**Table I:**
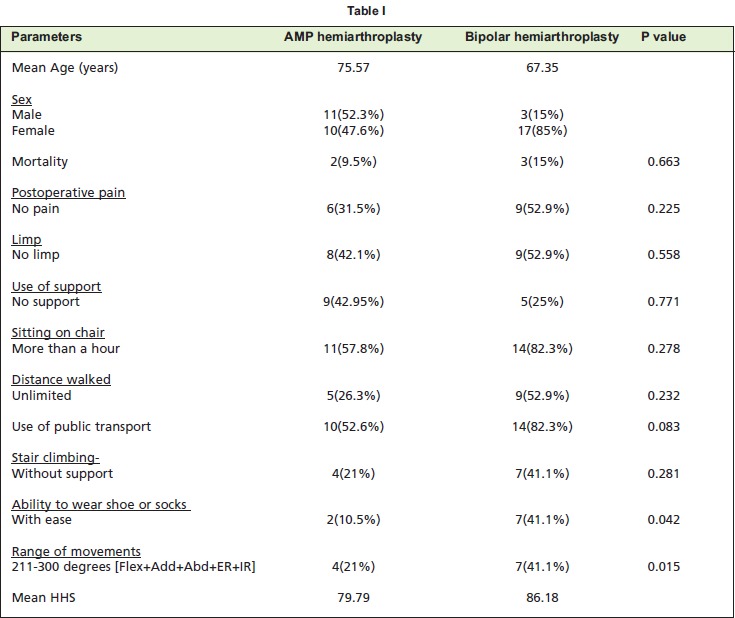


**Table II:**
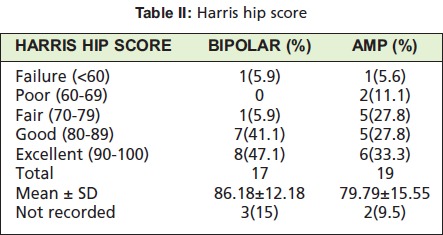
: Harris hip score

**Table III:**
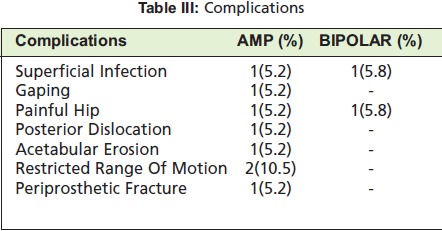
: Complications

## Conclusion

Which type of hemiarthroplasty should we select for the elderly patients with displaced fractures of the femoral neck? Based on the results of our study, there appears to be statistical difference between the two groups, that is bipolar being better in functional aspects. The results of our study showed that the incidence of complications were lower after bipolar hemiarthroplasty.

Some Western literature report the disadvantage of bipolar prosthesis as being more expensive but in our institution, there was not much cost difference between the two prostheses.
